# Wiregrass (*Aristida beyrichiana*) survival and reproduction after fire in a long-unburned pine savanna

**DOI:** 10.1371/journal.pone.0247159

**Published:** 2021-02-17

**Authors:** Jennifer M. Fill, Cesar Zamora, Carolina Baruzzi, Javier Salazar-Castro, Raelene M. Crandall

**Affiliations:** School of Natural Resources and Conservation, University of Florida, Gainesville, FL, United States of America; Brigham Young University, UNITED STATES

## Abstract

Restoring fire regimes is a major goal of biodiversity conservation efforts in fire-prone ecosystems from which fire has been excluded. In the southeastern U.S.A., nearly a century of fire exclusion in pine savannas has led to significant biodiversity declines in one of the most species-rich ecosystems of North America. In these savannas, frequent fires that support biodiversity are driven by vegetation-fire feedbacks. Understory grasses are key components of these feedbacks, fueling the spread of fires that keep tree density low and maintain a high-light environment. When fire is reintroduced to long-unburned sites, however, remnant populations of bunchgrasses might experience high mortality from fuel accumulation during periods of fire exclusion. Our objective was to quantify fire effects on wiregrass (*Aristida beyrichiana*), a key component of vegetation-fire feedbacks, following 16 years without fire in a dry pine savanna typically considered to burn every 1–3 years. We examined how wiregrass size and fuel (duff depth and presence of pinecones) affected post-fire survival, inflorescence and seed production, and seed germination. Wiregrass exhibited high survival regardless of size or fuels. Probability of flowering and inflorescence number per plant were unaffected by fuel treatments but increased significantly with plant size *(p* = 0.016). Germination of filled seeds was consistent (29–43%) regardless of fuels, although plants in low duff produced the greatest proportion of filled seeds. The ability of bunchgrasses to persist and reproduce following fire exclusion could jumpstart efforts to reinstate frequent-fire regimes and facilitate biodiversity restoration where remnant bunchgrass populations remain.

## Introduction

Fire regimes are important drivers of biodiversity patterns in fire-prone ecosystems [1]. In many regions of the world, variations in climate and human activity have caused fire regime shifts ranging from fire suppression to increased fire frequency and altered seasonality [2–4]. These changes have affected biodiversity in many ways, including shifts in plant functional groups [5] and animal species distributions [6]. Promoting biodiversity by restoring and maintaining fire regimes in flammable ecosystems, particularly where fires have been suppressed, is therefore a major goal of biodiversity conservation efforts [7].

Reintroduction of fire regimes in fire-prone pine savannas of the southeastern U.S.A. is a conservation priority. These ecosystems sustain an exceptional species richness and endemism [more than 50 species per m^2^; 8], making it crucial to restore the frequent ground fires (every 1–3 years) that maintain them [9–11]. Pine savannas are currently experiencing a long period of fire exclusion [12], which has led to visible changes in ecosystem structure and reductions in biodiversity [13]. A fundamental step towards their restoration is promoting vegetation-fire feedbacks [10,14], whereby flammable vegetation, such as grasses, fuel the spread of fires that keep midstory and overstory tree cover low. In turn, the high-light environment is conducive for the growth of understory vegetation that fuels future fires, resulting in a frequent fire regime (every 1–3 years). If fire is excluded, tree recruitment into the overstory increases, shading out the flammable understory and disrupting vegetation-fire feedbacks [14,15].

Restoration efforts often target grasses in the understory to support fire spread and reinstate vegetation-fire feedbacks. *Aristida beyrichiana* (wiregrass) is a perennial, endemic C4 bunchgrass that commonly dominates pine savanna understories, contributing to vegetation-fire feedbacks and biodiversity dynamics [16–18]. In savannas where fire has been excluded however, the size and number of wiregrass individuals are greatly reduced [19,20], which could be attributed to competition, lower light levels from overstory shading, or heavy litter [21–23]. Moreover, because sexual reproduction in wiregrass is primarily stimulated by fire, lack of fire inhibits wiregrass seed production [16]. Restoring populations of this dominant species and key component of pine savanna function is therefore a conservation and restoration challenge [24].

Although restoring frequent fire to pine savannas should increase biodiversity in the long-term, the initial state of the ecosystem could alter the restoration trajectory [25]. During long periods of fire exclusion, woody species typically increase in cover and shade out the understory, resulting in herbaceous species declines [23]. The fuel buildup (e.g., pine needle litter, hardwood leaves, pinecones) that results could increase the relative intensity and severity of a reintroduction fire. For example, long fire-free intervals allow fuels to accumulate, which can increase tree mortality through increased depth and duration of heating at the bases of trees [26]. Similarly, pine litter and duff accumulation around wiregrass plants could increase the amount and duration of heating and cause unusually high wiregrass mortality [27,28]. Previous studies suggest that wiregrass responses to fire after long, unburned periods should differ from responses to frequent return intervals. In a stand that had been fire-suppressed for at least 35 years, [13] found that wiregrass declined after a reintroduction fire and still had not reached pre-burn levels of cover after eight years. In addition, wiregrass’ inflorescence production has been shown to be lower after the reintroduction of fire in long-unburned areas relative to that in frequently burned areas [20], although it is unknown how this translates to seed viability and germination. None of these studies, however, examined the burning of accumulated fuels as a mechanism by which reintroduction fires affect wiregrass plants.

Our objective was to examine the effects of burning duff and pinecone fuels on remnant wiregrass populations when fire is reintroduced to long-unburned sites. We measured wiregrass plant size and quantified pinecone and duff fuels prior to a prescribed fire, and examined the fire’s effects on plant survival, inflorescence and seed production, and seed viability and germination. Weather conditions and burning techniques were favorable to cautiously achieving a low-intensity fire, which is typical of reintroduction fire objectives. We hypothesized that pinecones and deeper duff would increase plant mortality and result in decreased reproduction and seed germination. Because of wiregrass’ key role in vegetation-fire feedbacks, identifying mechanisms that could affect wiregrass survival and fecundity should help promote ecosystem and biodiversity restoration in pine savannas.

## Methods

### Study site

**Fig 1:**
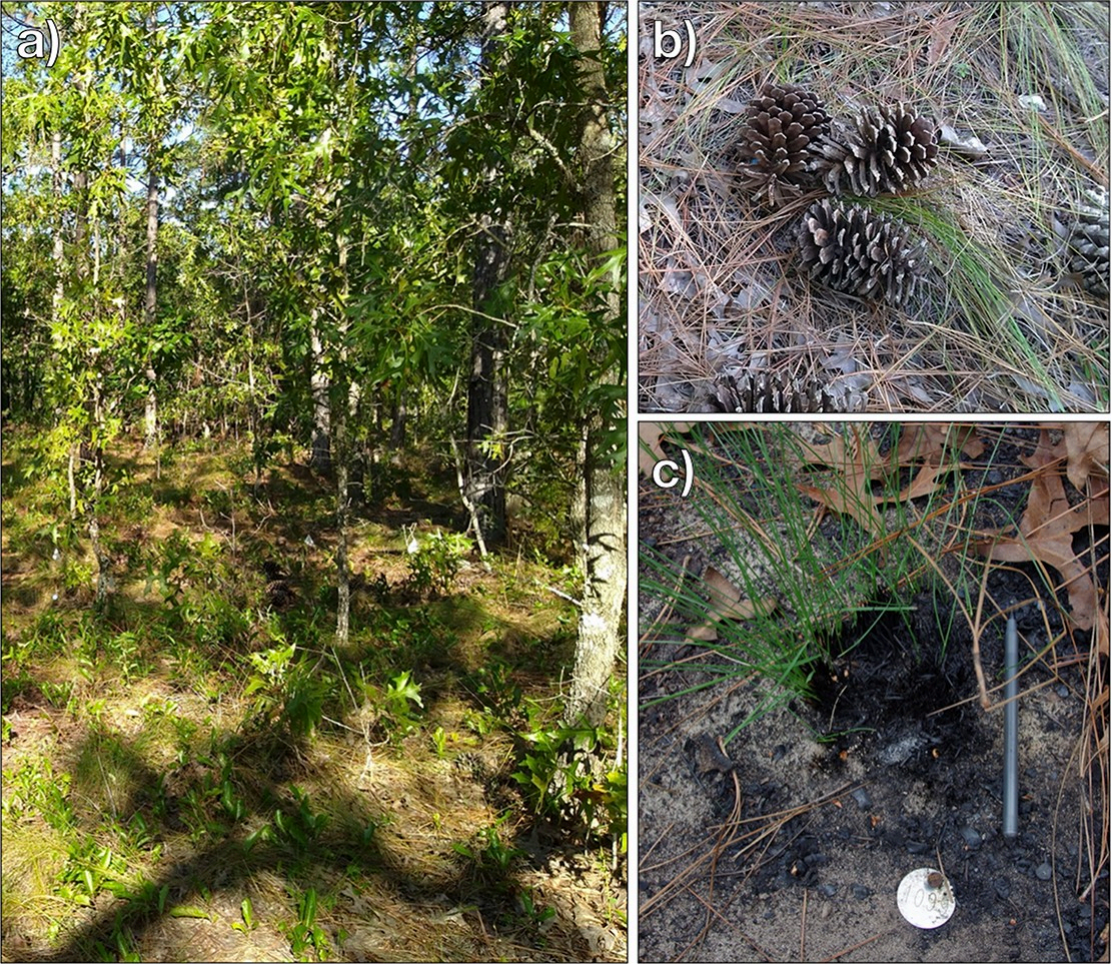
Study system and treatments. Wiregrass a) in the understory of a long unburned xeric pine savanna with an overstory dominated by longleaf pine and turkey oaks; b) under three pinecones for the low duff plus added pinecones treatment (LD + P); and c) after the reintroduction fire, with a portion of the plant resprouting (pen placed for size reference).

We conducted this study at the University of Florida Ordway-Swisher Biological Station in Putnam County, Florida (29°41’ N, 82°00’ W). The climate is humid, warm temperate with 20°C mean annual temperature and 1432 mm mean annual precipitation [29]. The site encompasses more than 3,700 hectares of lowland swamps and marshes and upland ecosystems that include xeric sandhill pine savannas, hammocks, mixed-pine hardwoods, and isolated wetlands. We conducted this experiment in a longleaf pine (*Pinus palustris*) sandhill savanna that was last burned in June 2003 (16 years prior; Fig 1A). The unit is located on excessively drained, Candler fine sand, 0–5 percent slopes, with an overstory dominated by longleaf pine, and the midstory dominated by hardwood trees, mainly turkey oak (*Quercus laevis*). The groundcover was composed of scattered wiregrass and pineywoods dropseed (*Sporobolus junceus*) and a continuous layer of duff (decomposed material between the intact litter layer and the soil surface) and fuels such as pine needles and hardwood leaves, interspersed with pinecones and small branches. This location would likely have experienced regimes of frequent (1–3 yr) natural fires, primarily in the late spring and early summer, coincident with increasing lightning frequencies and dry weather [12].

### Study species

Wiregrass is a warm-season, caespitose, C4 bunchgrass that is a major component of pine savanna understory communities in the southeastern Coastal Plain [12]. With meristems several centimeters or more below the soil surface [30] and thin leaves and aerated architecture [16], the species is highly flammable and regrows rapidly after fire [16,17]. Reproduction is primarily vegetative, although sexual reproduction can be stimulated by fire and other disturbances [16,31]. The species rarely flowers without fire, however, but even after fire not every plant will necessarily produce flowering culms. Wiregrass’ presence in areas subject to long periods without fire is evidence of its persistence and longevity.

### Field data collection

We haphazardly identified and tagged 104 wiregrass individuals across a range of sizes (i.e., 0.2 to 1387.9 cm^2^) spread over an area of about 0.1 km^2^. We considered an individual to be a compact unit of ramets at least 10 cm distant from other groups of ramets. Although we cannot verify that distance qualifies distinct genetic individuals, in this study we were concerned with linking aboveground recovery to fuels on those ramets, so our definition of “individual” is appropriate for this study. We calculated plant basal area as the area of an ellipse, using the longest axis of the base of the plant and the corresponding perpendicular axis. Each plant was tagged by pinning a uniquely numbered aluminum tag into the soil next to the individual. In demographic studies, the unit of replication is the individual on which vital rates (survival, reproduction) are measured, not the plot or burn unit level [32]. Replication at the unit level would have entailed burning another unit on a different day, in which case units would not have been true replicates owing to intra-day differences in weather conditions, human-ignition patterns, and fire behavior. Fires are heterogeneous across various scales, and thus is it is likely each of our replicate plants experienced different fires even though they were burned on the same day, providing random variation in fire characteristics across treatments.

Plants were categorized into three fuel treatments: high duff, low duff, and low duff with added pinecones. We measured duff depth as the average depth at four equidistant points around the base of each individual. To assign treatments, plants were first divided into low (<2 cm; n = 72; LD) or high (2–5 cm; n = 28; HD) duff categories based on duff depth around the base of each plant. We then ordered the database of plants in the low duff treatment by increasing size and assigned every third plant to have pinecones added to ensure we applied treatments across the full range of plant sizes (n = 26; LD+P); this left 46 plants in the low duff (LD) treatment without pinecones. We created the LD+P treatment to simulate heavy fuels that can potentially cause plant mortality (O’Brien et al. 2016) [27]. We did not add pinecones to plants in high duff because there were fewer individuals available in this category. Differences in litter depth between treatments were negligible. On the morning of the prescribed fire, three pinecones of approximately equal size were placed on top of plants assigned to the pinecone addition treatment (LD+P; Fig 1B). These pinecones had been collected from the stand and dried in a drying oven at 60°C for 48 hours just prior to the prescribed fire to eliminate the potential influence of differences in fuel moisture on outcomes. The unit was burned with a backing fire lit on 26 June 2019. Weather conditions and burn techniques were typical of those preferred when initially restoring prescribed fires to long-unburned pine savannas. Ten-hour fuel moisture was 15% and Keetch-Byram drought index (KBDI) was 218. Air temperature, relative humidity, and wind speed ranged from 30–35°C, 40–50%, and 3–5 km/h, respectively.

In November 2019, we surveyed plant survival and reproduction. For each reproductive plant, we counted all culms and measured the length of the inflorescence on every flowering culm. To quantify fecundity, we calculated the relationship between inflorescence length and number of glumes on 20 flowering culms and used this relationship to calculate number of seeds per plant.

In November 2019, we also collected seeds from plants by hand-stripping them from inflorescences. Seeds with their surrounding paleas and lemmas were obtained from individual plants, pooled by treatment (HD, LD, LD+P), and stored at 3°C for approximately one month prior to beginning germination experiments. In March 2020, we examined seed viability by separating filled seeds from empty and smut-infected seeds using the press test [33]. We counted seeds from each treatment, separating filled from empty and infected seeds until we obtained 96 filled seeds in each treatment. To examine germination of filled seeds, for each fuels treatment we placed 32 filled seeds, including palea and lemma, on blotter paper in each of three polystyrene boxes (total n = 96) to which we added 5 mL of a 0.002% solution of Plant Preservative Mixture (https://www.plantcelltechnology.com/about-ppm/). The boxes were placed on a lab bench at room temperature. We periodically monitored and removed germinated seeds in each box and ended the experiment after two months. We also placed the empty and infected seeds in similarly prepared boxes to confirm our seed quality assessment.

### Statistical analysis

Because nearly all plants survived, it did not make sense to statistically analyze survival. We analyzed reproduction data from 99 surviving plants across all sizes (one surviving individual from the low duff + pinecones treatment could not be relocated). To investigate whether fuel treatment (HD, LD, LD+P) affected the probability of reproduction, we used logistic regression with basal area and fuels treatment as predictors of whether a plant produced flowering culms. The Hosmer-Lemeshow statistic was not significant for probability of reproduction (*p* = 0.36), indicating good model fit. To investigate whether fuel treatments affected seed production, we used the 77 plants which produced flowering culms. Because the number of culms per plant was correlated with the number of seeds (Spearman’s rank correlation coefficient = 0.97), we used a generalized linear model to analyze the number of culms per plant only, with basal area and fuel treatment as predictors. We used negative binomial regression (MASS package; [34]) after testing the overdispersion of the number of culms data with respect to a Poisson distribution by comparing the residual deviance to the residual degrees of freedom [35]). We used LD as the reference level for fuel treatment because this represented the lowest fuel level, in contrast to increased fuels (HD and LD+P). A comparison of the full model for culm production to the null model was significant, indicating that the predictors improved the model.

We analyzed seed germination data using logistic regression on the probability of germination (lme4 package; [36]). We used the status of filled seeds (i.e., germinated, non-germinated) at the end of the trial period as the response variable and fuels treatment as predictor. There was not enough variation among boxes to include germination box as a random effect. The Hosmer-Lemeshow statistic was not significant for probability of germination (*p* = 1). All analyses were conducted in R [37].

## Results

Wiregrass plants exhibited low overall mortality regardless of size or fuel treatment. Of the 104 plants burned across the three treatments, all but four individuals survived. Three plants were in the LD+P treatment, and one was in the LD treatment. All were small plants (<15cm^2^).

**Fig 2:**
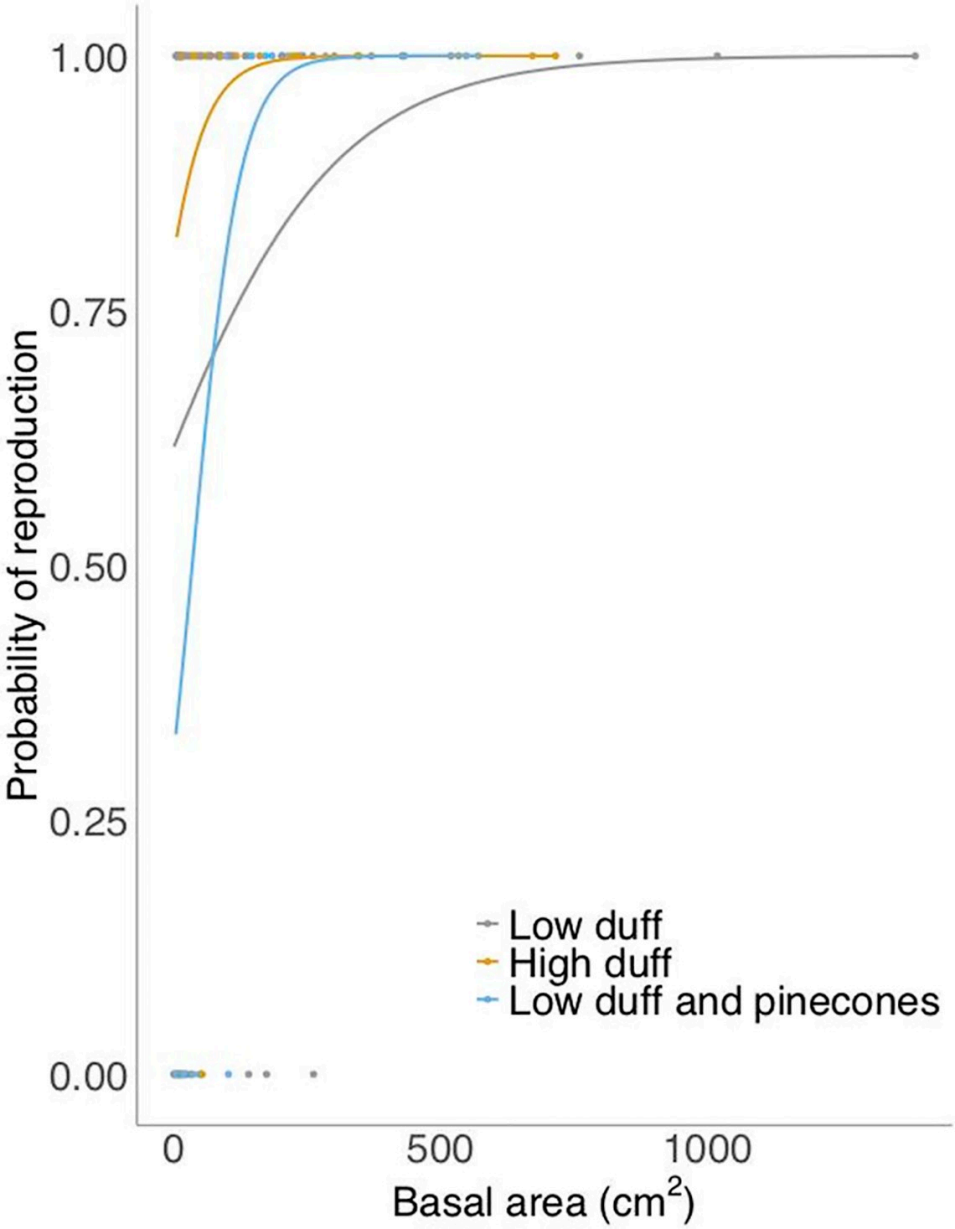
Probability of reproduction (inflorescence production) by wiregrass (*Aristida beyrichiana*) plants. Individuals were subject to different fuel treatments during a reintroduction fire in a long-unburned pine savanna. Probability of reproduction increased with size. There were no significant differences between individual treatments.

Wiregrass fecundity varied by size. The probability of reproduction significantly increased with basal area (Table 1), with plants greater than 200cm^2^ more likely to be reproductive (Fig 2). In reproductive plants, the number of culms per plant also increased significantly with basal area, regardless of fuel treatment (Table 1). The germination of filled seeds did not differ between fuel treatments (Table 1). The percentage of filled seeds that germinated in HD, LD, and LD+P treatments was 29, 43, and 34, respectively. The total number of empty or infected seeds in HD, LD, and LD+P treatments was 172, 94, and 171 (or 64%, 49%, and 64% of the number of seeds sampled), respectively. Very few empty/infected seeds germinated, confirming our seed quality categorization (filled/empty); there were no differences between treatments (*p*>0.400).

**Table 1:** Post-fire reproduction and germination.

	Coefficient	Standard Error	*P*	Odds Ratio
Probability of reproduction^*a*^				
Intercept	0.23	0.42	0.573	1.26
Basal area	0.01	0.00	0.016	1.01
High duff	1.61	0.83	0.052	5.00
Low duff + pinecones	-0.34	0.58	0.565	0.71
Culms per plant^*b*^				
Intercept	2.37	0.23	0.000	
Basal area	0.002	0.001	0.000	
High duff	-0.55	0.30	0.073	
Low duff + pinecones	-0.10	0.34	0.762	
Probability of germination^*a*^				
Intercept	-0.30	0.21	0.155	0.75
High duff	-0.60	0.31	0.052	0.55
Pinecone addition	-0.35	0.31	0.236	0.70

Regression model results for reproduction and germination of wiregrass (*Aristida beyrichiana*) plants under different fuel treatments after a reintroduction fire in a long-unburned pine savanna.

^*a*^ Logistic regression model.

^*b*^ Generalized linear model.

## Discussion

High survival of wiregrass coupled with its fecundity after fire suggests that burning after nearly two decades without fire should promote population recovery. Nearly all plants survived regardless of fuels. Under typical field conditions, pinecones are likely have at least some moisture that could result in lower fuel consumption and heat transfer, so our results are likely a conservative estimate of survival. After our experimental fires, we documented some surviving individuals with only a portion of the pre-fire plant resprouting (Fig 1C). It is possible that surviving wiregrass individuals are able to persist even with duff and pinecones because of small-scale fire heterogeneity, more numerous buds, or more resources to allocate to regrowth after fire.

Wiregrass survival could be significantly affected by fuels in long-unburned stands where litter accumulation is much greater than at our experimental site, such as after even longer periods of fire exclusion. Heavy fuel loads have been shown to affect plant survival through increased fire intensity and longer durations of flaming and smoldering [17,27,38], which should increase the likelihood of heat transfer to greater soil depths [39] where wiregrass apical buds are located. Pine needle fuel loads of 2 kg/m^2^ can generate fire temperatures of up to 1000°C [40], and pinecones can smolder for more than 30 minutes at 400°C [41]. Although wiregrass has been shown to resprout after fires with max temperatures as high as 500°C [42], the threshold fire intensity and heating duration above which individuals do not survive is unknown but would likely depend on soil heating patterns within the entire plant.

Wiregrass fecundity was affected by pre-fire size but not by fuel treatment. Larger plants were more likely to reproduce and to produce more flowering culms than smaller plants. Increased reproductive output with size has been previously documented in this species [43,44] and in other pine savanna bunchgrasses [45,46]. Although exposure to high fire temperatures has been shown to stimulate greater wiregrass culm production (500°C for 12 minutes; 42), our pinecone addition treatment might not have been sufficiently intense or of sufficient heating duration to stimulate an increase in fecundity. Germination of filled seeds was similarly unaffected by fuel treatment and was high across all treatments. Early to mid-summer (May-July) fires in the region, such as the prescribed fire in this study, tend to increase production of viable seeds relative to spring (February-March) or late summer (August) fires [42,43]. Plants in the LD treatment, however, produced twice as many filled seeds as plants burned under greater fuel loads. This trend suggests a tradeoff between culm production [42] and production of potentially germinable seeds with variation in fire intensity/severity.

Environmental conditions and duration of fire exclusion influence the process of re-establishing the vegetation-fire feedback and facilitating biodiversity recovery. In more mesic pine savannas, there are significant increases in shrub and litter cover and reductions in relative cover and frequency of herbaceous species between 13- and 25-years post-fire [19]. In mesic pine savannas that had been fire-suppressed for more than 35 years, wiregrass did not recover to pre-burn levels after 8 years but exceeded pre-burn levels of cover within 4 years at a more recently burned site (fewer than 20 years since fire; 13). In a xeric pine savanna, [47] found that wiregrass and other grasses increased significantly after three reintroduction fires over a ten-year period, following 63 years of fire exclusion. Dry pine savanna sites might be more resilient to fire exclusion than wetter or more fertile locations, particularly with lower vegetation growth rates and slower rates of litter accumulation [48]. Thus, as time-since-fire increases, the likelihood of recovering species biodiversity declines, especially in wetter sites. If the threshold for passively re-establishing vegetation-fire feedbacks through reintroducing fire is relatively longer in drier sites, our study site might not have crossed this threshold.

Restoration of fire-excluded pine savannas to a frequently burned, biodiverse condition depends on reestablishing vegetation-fire feedbacks. As one of the dominant groundcover grasses over much of the range of pine savannas [16,24], wiregrass appears to be resilient to lack of fire in dry environments for at least 20 years. The ability of this species to persist and reproduce following long periods without fire could jumpstart efforts to reinstate a frequent-fire regime where remnant populations remain, which should facilitate biodiversity restoration in fire-excluded sites.

## Supporting information

S1 Table Data on size and survival of wiregrass (*Aristida beyrichiana*) plants under different fuel treatments after a reintroduction fire in a long-unburned pine savanna. (CSV)

S2 Table Data on reproduction of wiregrass (*Aristida beyrichiana*) plants under different fuel treatments after a reintroduction fire in a long-unburned pine savanna. (CSV)

S3 Table Data used for analyses of wiregrass (*Aristida beyrichiana*) seed germination. (CSV)
